# Normative age-related structural brain deviations underlying psychopathology, cognitive impairment and neurological soft signs in schizophrenia spectrum disorders

**DOI:** 10.1038/s41398-026-03956-0

**Published:** 2026-03-20

**Authors:** Sebastian Volkmer, Katharina M. Kubera, Stefan Fritze, Geva Brandt, Dilsa Cemre Akkoc Altinok, Jonas Daub, Jacqueline Kukovic, Kent-Tjorben Böttcher, Oksana Berhe, Yuchen Lin, Heike Tost, Andre F. Marquand, Andreas Meyer-Lindenberg, Emanuel Schwarz, Dusan Hirjak

**Affiliations:** 1 Department of Psychiatry and Psychotherapy, Central Institute of Mental Health, Medical Faculty Mannheim, University of Heidelberg, Mannheim, Germany; 2 Hector Institute for Artificial Intelligence in Psychiatry, Central Institute of Mental Health, Medical Faculty Mannheim, Heidelberg University, Mannheim, Germany; 3 German Centre for Mental Health (DZPG), Partnersite Mannheim-Heidelberg-Ulm, Mannheim, Germany; 4 Center for Psychosocial Medicine, Department of General Psychiatry, University of Heidelberg, Heidelberg, Germany; 5 Donders Centre for Cognitive Neuroimaging, Donders Institute for Brain, Cognition and Behaviour, Radboud University, Nijmegen, the Netherlands; 6 Department of Cognitive Neuroscience, Radboud University Medical Centre, Nijmegen, the Netherlands

**Keywords:** Pathogenesis, Neuroscience

## Abstract

Schizophrenia spectrum disorders (SSD) are marked by widespread structural brain abnormalities. Neuroanatomical normative modeling (NM) can quantify person-specific deviations from healthy variability, yet it remains unknown whether pre-trained, large-scale NM features support site-held-out classification and mechanistic brain–behavior mapping in SSD. Here, we applied a publicly available PCNtoolkit model (trained on ~57,000 healthy controls from 82 sites) to six independent cohorts (N = 831) to derive individual deviations in cortical thickness (CT) and subcortical volumes from T1-weighted MRI. Employing a random forest classifier with leave-site-out cross-validation, we achieved a balanced accuracy of 65%, which underscores the inherent complexity of SSD. Feature importance analysis identified total gray matter volume, mean CT, and CT changes in limbic and sensorimotor regions as key predictive features. Relative to healthy controls, SSD participants showed a higher burden of extreme negative deviations, which related to reduced attention and processing speed and to elevated neurological soft signs (NSS). Finally, canonical correlation analysis revealed a robust multivariate relationship linking structural deviationsparticularly CT changes in limbic and sensorimotor cortices, to cognition and NSS. Together, these results demonstrate that NM features transferred from a large external reference can generalize across sites and elucidate clinically relevant brain–behavior associations in SSD, supporting the integration of multimodal, large-scale datasets to advance biomarker discovery and inform earlier, more targeted interventions.

## Introduction

Schizophrenia spectrum disorders (SSD) have a strong neurodevelopmental [1] component and an often chronic or relapsing course, underscoring the importance of a lifespan approach to their pathophysiology. While SSD present widespread structural brain changes, pinpointing consistent neuroanatomical alterations has been difficult due to the disorder’s marked heterogeneity [2–4]. Numerous cross-sectional and longitudinal studies have documented these structural abnormalities [5] but their high variability has hindered the establishment of reliable, reproducible patterns, complicating efforts to definitively implicate or rule out specific cortical and subcortical regions [6–10]. Moreover, it is hypothesized that SSD encompasses additional neurodegenerative [11] processes, adding to the disorder’s complexity [12, 13]. These challenges underscore the need for analytical approaches that can inform us about neurodevelopmental trajectories and disease progression at the single-subject level. Situated within the framework of precision medicine, normative modeling (NM) is an emerging and promising framework for mapping individual differences across the lifespan in relation to a reference model [14]. This means that an individual can be located within the normative distribution to establish to what extent they deviate from the expected pattern in distinct neuroimaging measures, and a map can be generated of where and to what extent an individual’s brain differs from the reference norm [15, 16].

Normative models are trained on extensive neuroimaging datasets derived from healthy cohorts across multiple study sites, enabling precise computation of deviations across diverse brain regions. Among the various NM frameworks available, one widely used approach is the Predictive Clinical Neuroscience toolkit (PCNtoolkit) [17]. For this study, we utilized the PCNtoolkit model, available on its official GitHub repository (https://github.com/amarquand/PCNtoolkit). This model was trained on Freesurfer 6.0.0 data from a large reference cohort comprising approximately 57,000 healthy control (HC) pooled from 82 distinct study sites. While neuroimaging-based brain age and NM have been proposed as a framework for biomarker identification in SSD [18], their utility for cross-site machine learning (ML) applications under strict generalization (e.g., leave-site-out validation) remains insufficiently characterized.

Although accelerated brain aging can be detected through various neuroimaging methods in SSD [19], the direct connection to the mechanisms underlying cognitive or sensorimotor deficits is not well understood. Several NM studies have been conducted in SSD and related psychotic disorders, and some have examined SSD alongside other mental disorders such as attention-deficit/hyperactivity disorder (ADHD), autism-spectrum disorders (ASD), or bipolar disorder [20–25]. Investigations using structural T1-weighted MRI, diffusion MRI, and functional MRI have reported widespread yet heterogeneous deviations [2, 22, 23, 26], and only two have probed NM’s predictive power—one using diffusion MRI [24] and the other combining T1-weighted and resting-state fMRI [23]. Prior findings indicated that deviations from normative brain structure are widespread in SSD [2, 14, 21, 24, 27], spanning white matter tracts and cortical thickness (CT), only about 15–20% of patients exhibit significant aberrations in any single region. Yet, a striking 79% show at least one infranormal deviation, underscoring the profound heterogeneity inherent to SSD [28]. At the same time, links between individual deviation profiles and behavior (e.g., psychopathology, cognition, and neurological soft signs (NSS)) remain incompletely understood, particularly using multivariate brain–behavior frameworks.

To address the issue of heterogeneous presentation of SSD and its neurodevelopmental and neurodegenerative component, we analyzed a combined sample of 831 subjects—522 healthy controls and 309 SSD participants—drawn from four independent in-house and two publicly available cohorts (UCLA [29] and COBRE [30]). Our investigation pursued four key objectives: First, to test whether NM features transferred from a large, external reference model support generalizable (leave-site-out) classification using a Random Forest (RF) classifier. Second, we assessed the RF model’s feature importance using Mean Decrease in Impurity (MDI) metric, aiming to identify robust, cross-site markers of brain deviations and their associations with psychopathological symptoms, sensorimotor alterations, and cognitive functioning. Third, we employed Canonical Correlation Analysis (CCA) to unravel the multivariate relationships between deviations in CT and various behavioral measures, including psychopathological symptoms, sensorimotor deficits, and cognitive performance. By bridging the gap between brain structure and behavior, our study seeks to illuminate the neurobiological mechanisms underpinning schizophrenia. Finally, we used a summary score typically used in the analysis of NM, i.e., the count of extreme negative deviations, to assess its relation to these psychopathological, cognitive and sensorimotor domains. By uniting these multi-dataset analyses, advanced machine-learning methods, and the examination of both individual deviations and summary measures, our findings clarify the multifaceted neurobiology of schizophrenia, linking cortical abnormalities to key behavioral domains and setting the stage for more personalized, predictive clinical approaches.

## Methods

### Data

#### Study participants within the inhouse datasets

This study incorporated four distinct in-house datasets, hereafter referred to as whiteCAT, URBN, NSS, and BMBF. Written informed consent was obtained from both SSD patients and HC following a comprehensive explanation of the study procedures. Participants diagnosed with SSD were assessed during in- or outpatient treatment shortly after achieving partial remission of acute psychopathological symptoms. All key study procedures, including psychopathological evaluations and neuropsychological assessments, were conducted within seven days. Patients were maintained on a stable daily dose of antipsychotic or antidepressant medication for at least seven days prior to inclusion in the study.

Since the whiteCAT and NSS cohorts included only patient data, while the BMBF and URBN cohorts consisted solely of HC, we pooled these datasets for classification. Specifically, we merged whiteCAT with URBN and NSS with BMBF, as each pair was acquired on the same scanner using an identical imaging protocol. This resulted in two independent datasets consisting of SSD patients and HC.

The severity of psychopathological symptoms in SSD patients was evaluated using the Positive and Negative Syndrome Scale (PANSS) [31], which includes subscales for positive, negative, and general symptoms. Sensorimotor alterations were assessed using the Heidelberg NSS scale (H-NSS) [32] in SSD. The H-NSS covers five different subscales called motor coordination (MOCO), integrative functions (IF), complex motor tasks (COMT), right/left spatial orientation (RLSPO), and hard signs (HS). Cognitive functioning was evaluated using the Brief Cognitive Assessment Tool (B-CATS) [33] (for demographic and clinical details see Tables 1 and 2).

**Table 1:** Demographic characteristics of in-house and publicly available datasets that were used in the analysis.

Study	HC/SSD	Sex (M/F)	Age	Dropped QC (Before/After Preprocessing)
whiteCAT	0/74	52/22	36.77 (SD=12.94)	6/0
URBN	74/0	50/24	26.84 (SD=7.1)	0/1
NSS	0/112	61/51	38.6 (SD=11.48)	4/13
BMBF	220/0	115/105	27.27 (SD=9.19)	0/1
UCLA	30/30	48/12	36.02 (SD=9.09)	34/8
COBRE	74/74	119/28	37.86 (SD=12.71)	20/3

This table does not include adaption data. The adaption data includes addtional whiteCAT/URBN n = 25; NSS/BMBF n = 25; UCLA n = 78, COBRE n = 14.

**Table 2:** Clinical characteristics of in-house datasets consisting schizophrenia spectrum disorders patients (n = 186).

Clinical Parameter	whiteCAT n = 74	NSS n = 112	Mann-Whitney U test (p-values)
Duration of Illness	10.69 (SD=10.19)	10.31 (SD=10.76)	n.s.
PANSS Total score	65.4 (SD=16.16)	67.42 (SD=21.46)	n.s.
PANSS Positive score	15.89 (SD=5.66)	15.65 (SD=6.97)	n.s.
PANSS Negative score	16.76 (SD=6.84)	16.69 (SD=7.37)	n.s.
PANSS General score	32.79 (SD=7.66)	35.17 (SD=10.99)	n.s.
NSS Total score	19.96 (SD=8.43)	18.93 (SD=8.2)	n.s.
NSS Motor Coordination	7.17 (SD=4.21)	7.47 (SD=3.8)	n.s.
NSS Integrative Functioning^b^	4.46 (SD=1.85)	2.6 (SD=1.5)	>0.001
NSS Complex motor tasks	3.33 (SD=1.81)	3.37 (SD=2.25)	n.s.
NSS Spatial Orientation^a^	3.51 (SD=2.77)	2.56 (SD=2.38)	0.02
NSS Hard Signs	3.28 (SD=2.14)	2.93 (SD=1.75)	n.s.
TMT-B	100.99 (SD=63.73)	111.72 (SD=65.72)	n.s.
DSST^b^	59.04 (SD=19.62)	49.88 (SD=26.87)	0.005
CF	20.65 (SD=6.58)	18.43 (SD=13.12)	n.s.

The clinical battery consisting of PANSS, NSS and cognitive tests were collected on SSD patients (whiteCAT and NSS cohorts) only.

*PANSS* Positive and Negative Syndrome Scale; *NSS* Neurological Soft Signs; *DSST* Digit Symbol Substitution Test; *TMT-B*, Trail Making Test B; *CF* Categorical Fluency.

^a^ Indicate variables that are significantly different between datasets using Mann-Whitney U tests.

^b^ Indicate variables that survive the FDR correction. Significant differences between the datasets were found in DSST (U = 4950.5, p = 0.005, q = 0.04; rank-biserial r = −0.25) and NSS Integrative (U = 6255.0, p = 3.26 × 10⁻¹¹, q = 4.57 × 10⁻¹⁰; r = −0.57). The NSS Spatial subscore was nominally significant (U = 4785.0, p = 0.02, r = −0.02) but did not survive FDR correction (q = 0.09).”

The whiteCAT study included 80 SSD patients recruited from the in- and outpatient departments of the Central Institute of Mental Health (CIMH) in Mannheim, Germany, as part of a larger cohort (see Hirjak et al. for the study rationale [34]). Psychiatric diagnoses were clinically evaluated by GAB, SF, and DH. The study was approved by the local Ethics Committee II (Medical Faculty Mannheim at Heidelberg University, Germany), enrolled adults aged 18–64 with ICD-11 diagnoses of schizophrenia or other primary psychotic disorders (codes: 6A20–6A25), with or without psychotic symptoms. Exclusion criteria included inability to speak German, known mental retardation (IQ < 70) or dementia, substance use disorder in remission for less than 12 months, and neurological or medical conditions affecting the constructs being assessed. The URBN study enrolled HC (19–53 years) at CIMH, Mannheim, Germany, with participants a priori assigned to training (n = 75) and validation (n = 25) cohorts. Individuals with any prior diagnosis of a SSD were excluded following screening.

The NSS study [35] included 129 subjects who met DSM-IV criteria for SSD. Diagnoses were made by staff psychiatrists and confirmed using the German versions of the Structured Clinical Interview for DSM-IV Axis I and II Disorders (SCID) and examination of case notes (reviewed by SF and DH). The BMBF study [36] enrolled 246 healthy controls (HC; 18–58 years) at the CIMH, Mannheim, Germany, with participants a priori assigned to training (n = 221) and validation (n = 25) cohorts; individuals with any prior schizophrenia spectrum disorder (SSD) diagnosis were excluded at screening.

#### Participants within the publicly available data

The UCLA multimodal dataset, sourced from the LA5c study conducted by the UCLA Consortium for Neuropsychiatric Phenomics, includes multimodal MRI images from 272 participants [29]. These participants are either HCs (N = 130) or individuals diagnosed with mental illnesses, including SSD (N = 50). Participant ages range from 21 to 50 years. The dataset was obtained from the OpenfMRI database (https://openfmri.org/dataset/ds000030/) and is identified by the accession number ds000030 [37]. HC were recruited through community advertisements in Los Angeles and were required to have at least 8 years of education, be fluent in English or Spanish, and free of major psychiatric disorders such as schizophrenia, bipolar disorder, ADHD, substance abuse, or major depression. In contrast, individuals with schizophrenia, who met the same age and health criteria, were recruited via targeted clinical outreach and online platforms. All participants provided written informed consent under protocols approved by UCLA and the Los Angeles County Department of Mental Health, and those undergoing fMRI sessions were further screened for safety.

The COBRE dataset, sourced from the Center of Biomedical Research Excellence (COBRE) [38], was accessed via https://schizconnect.org. It includes sMRI data from HCs (N = 91) and participants with SSD (N = 85). The dataset comprises 176 participants aged 18 to 66 years. Participants were excluded for neurological disorders, head trauma (loss of consciousness >5 min), or substance abuse within the preceding 12 months. Diagnoses were establieshed using the Structured Clinical Interview for DSM Disorders. The SSD comprised schizophrenia (N = 74) and schizoaffective disorders (N = 11) [30]. Other labels such as bipolar disorder did not meet our inclusion criteria (for demographics see Table 1).

#### MRI data acquisition within the in-house datasets

Both whiteCAT and URBN studies involved sMRI scanning at CIMH using a 3 Tesla MAGNETOM Prisma MR scanner (Siemens Medical Solutions, Erlangen, Germany), with structural MRI conducted via T1-weighted 3D magnetization prepared rapid gradient echo sequence (MP-RAGE) parameters: 192 sagittal slices, image matrix = 256 × 256 mm², voxel size = 1 × 1 × 1 mm³, TR = 2,300 ms, TE = 3.03 ms, TI = 900 ms, and flip angle = 9°.

Both NSS and BMBF studies included structural MRI scanning at CIMH on a 3.0 Tesla Magnetom TIM Trio MR scanner (Siemens Medical Systems, Erlangen, Germany) equipped with a 32-channel multiarray head coil. T1-weighted 3D MP-RAGE data were acquired with the following parameters: 176 sagittal slices, field of view = 256 × 256 mm², voxel size = 1 × 1 × 1 mm³, TR = 2,530 ms, TE = 3.8 ms, TI = 1,100 ms, and flip angle = 7°.

#### MRI data acquisition within the publicly available datasets

For the UCLA study the structural MRI data was measured on one of two 3 T Siemens Trio scanners, at Ahmanson-Lovelace Brain Mapping Center and Staglin Center for Cognitive Neuroscience at UCLA. The MP-RAGE was acquired with the following parameters: 176 sagittal slices, field of view = 256 × 256 mm², voxel size = 1 × 1 × 1 mm³, TR = 1.9 s, TE = 2.26 ms.

Data acquisition was facilitated by the Collaborative Informatics and Neuroimaging Suite Data Exchange (COINS; http://coins.mrn.org/dx). Collection took place at the Mind Research Network and was supported by an NIH Center of Biomedical Research Excellence (COBRE) grant (5P20RR021938/P20GM103472), awarded to Dr. Vince Calhoun. The structural MRI data was collected on a Siemens 3 T TIM Trio scanner located at the MRN. The MP-RAGE was acquired with the following parameters: field of view = 256 × 256 mm², voxel size = 1 × 1 × 1 mm³, TR = 2.53 s, TE = [1.64, 3.5, 5.36, 7.22, 9.08 ms], TI = 1,200 ms, and flip angle = 7°.

### MRI-Preprocessing

All MRI data were preprocessed using fMRIPrep version 23.2.3 [39]. Intensity non-uniformity (INU) in the T1-weighted (T1w) images was corrected using N4BiasFieldCorrection [40], implemented in ANTs version 2.5.0 [41], and the resulting T1w image served as the reference throughout the preprocessing workflow. Skull stripping of the T1w reference image was performed using a Nipype implementation of the ANTs antsBrainExtraction.sh workflow, with the OASIS30ANTs template as the target. Segmentation of brain tissue into cerebrospinal fluid (CSF), white matter (WM), and gray matter (GM) compartments was conducted on the skull-stripped T1w images using the FAST algorithm [42]. Cortical surface reconstruction was carried out with recon-all (FreeSurfer version 7.3.2 [43]).

#### Calculating normative deviations

To estimate CT and subcortical volume, we processed neuroimaging data using FreeSurfer version 7.3.2 [43]. We then employed the lifespan_57K_82sites model from the PCNToolkit package to calculate normative deviations for each brain feature (CT and subcortical volumes) [17]. To reduce potential biases associated with larger or smaller adaptation sets, we adapted the publicly available normative model for each of our four sites/datasets independently. Accordingly, we adapted four separate models: one using the COBRE dataset, one using the UCLA dataset, one using the combined whiteCAT–URBN datasets, and one using the combined NSS–BMBF datasets. Before computing normative deviations, we adapted the model using HC (whiteCAT/URBN n = 25; NSS/BMBF n = 25; UCLA n = 78; COBRE n = 14) within each dataset to improve its relevance to the respective population. PCNToolkit relies on hierarchical Bayesian regression to account for site effects, the method is described in previous papers [23, 44]. For the in-house datasets, specifically, we incorporated small validation sets comprised of HC from the URBN and BMBF cohorts to further refine the model’s adaptation. For the COBRE and UCLA dataset, we utilized a propensity score matching using calculated logistic propensity scores with k-nearest-neighbor matching algorithm. HC were matched based on age and sex given the data availability [45]. HC that were not selected during propensity score matching were instead used for fine-tuning or adapting the NM. As highlighted by Barkema et al. [46], a subset of HC independent from the analysis sample should be utilized for model adaptation. This step was crucial to ensure that scanner-related variability did not confound the identification of structural deviations in SSD, thereby enhancing the reliability of our findings. Importantly, age and sex were included as covariates in all calculations to account for their influence on brain structure as described in the example “Braincharts: transfer” of the PCNToolkit.

#### MRI-Quality-Control

SV, KMK, and DH carried out the manual inspection of all MRI data. First, a quality control (QC) of the raw images, before any preprocessing steps, was carried out. Here, we mainly investigated certain motion artefacts, scanner effects, or a bad image quality, e.g., strong scanner inhomogeneity. Second, a QC was performed on the processed MP-RAGE images by investigating the HTML reports of fMRIPrep to investigate the quality of gray- and white matter boundaries. Since the UCLA has a known effect of ghosts in their brain scans, all images with the ghost-tag are previously dropped. This resulted in the following dropouts, 0: whiteCAT, 1: URBN, 13: NSS, 4: BMBF, 8: UCLA, and 3: COBRE.

### Statistical analyses

For our initial ML analysis, we employed a RF algorithm implemented via the scikit-learn Python package [47]. We utilized nested leave-site-out CV across all four datasets (N = 688, SSD N = 290, HC N = 398), training the model on three datasets, including hyperparameter optimization, while validating it on the excluded dataset. For hyperparameter optimization we used grid search on the inner loop, with the following ranges of each hyperparameter: number of estimators = [50, 100, 200]; maximum number of features = [0.3, 0.5, 0.7]; minimum number of samples per leaf = [1,2,4]. To address class imbalances, we applied the Synthetic Minority Over-sampling Technique (SMOTE) [48], followed by scaling the data using the StandardScaler from scikit-learn to ensure consistent feature distributions. Model performance was evaluated through accuracy, balanced accuracy, F1 score, precision, and the area under the receiver operating characteristic curve (AUROC). To compare ourselves with previous works we additionally compute the previously mentioned test scores on the combined cohort with 10-fold CV [23].

To further investigate feature importance, we trained a RF model on all of the four inhouse datasets and two external datasets and assessed the Mean Decrease in Impurity (MDI) metric as implemented in scikit-learn. MDI is a tree specific feature importance measure and for each feature is summed across all trees in the forest every time the feature is used to split a node. The higher the MDI value is, the more important is this feature to make the SSD prediction. To account for the imbalanced size of the datasets we fixed the size of the smallest dataset (UCLA n = 60) and performed bootstrapping (n = 10,000) to randomly select 60 subjects from each of the other datasets, i.e., COBRE whiteCAT/URBN, and NSS/BMBF, and took the mean MDI across all 10,000 bootstraps. We additionally counted the number of times during the leave-site-out CV where the features were among the top 20 most predictive features, resulting in a number from 1–4 to further ensure the robustness of features. The top 20 features based on the MDI, and a CV-count of at least 2 were selected for subsequent analyses. Model settings of the RF in this iteration was based on the hyperparameter combination that was most frequently selected during the nested-CV, i.e., number of estimators = 200, maximum number of features = 0.7, and minimum number of samples per leaf = 4.

To evaluate how cross-site generalizable brain features identified by the previously described ML approach relate to sensorimotor, cognitive, and psychopathological dimensions, we performed two analyses in our clinical inhouse SSD-cohorts (N = 186, whiteCAT and NSS). First, we conducted univariate analyses to examine associations between identified brain deviations (top 20) and behavioral parameters, including PANSS total score and three subscores, NSS total score and five subscores, and cognitive test scores from the B-CATS. Pearson correlations were computed, and the resulting p-values were corrected for multiple comparisons using the Benjamini-Hochberg false discovery rate (FDR) method [49]. From here, corrected p-values are referred to as q-values. To address whether significant correlations were driven by outliers, we performed a sensitivity analysis restricted to FDR-significant pairs. We report Spearman’s ρ as a robust alternative to Pearson’s r and quantify single-observation dependence via leave-one-out ranges in which the Pearson correlations are recomputed. Lastly, we recomputed Pearson’s r after excluding observations with Cook’s D > 4/n. Second, we conducted a CCA to assess multivariate associations, incorporating duration of illness (DOI) while excluding the total scores for NSS and PANSS to avoid inflating the empirical p-value generated via permutation testing. We focused on the first canonical correlation, as it represents the strongest multivariate relationship [50]. Loadings for each feature were correlated with their corresponding brain and behavioral parameters. To evaluate generalizablity, we performed 1,000 permutations to derive the empirical p-value and confirm the significance of the canonical correlation. We additionally, calculated the out of site prediction of the canonical variates, by calculating the CCA on one site and validate it on the other site (i.e., whiteCAT and NSS).

To characterize heterogeneity, we counted extreme negative deviations per participant across regions, defining extremes as z < −1.96. Group differences (HC vs SSD) in the per-subject extreme count were tested using generalized estimating equations (GEE; binomial link) with site as the clustering factor and age and sex as covariates.

Associations between the extreme-count and behavioral measures were examined in participants with complete behavioral data (N = 184, whiteCAT and NSS) using linear mixed-effects models with site as a grouping (random-effects) factor and age and sex as covariates. P-values were FDR-corrected across outcomes.

All above mentioned methods were performed in accordance with the relevant guidelines and regulations.

## Results

### Demographic and clinical characteristics

Demographic characteristics of all study cohorts are shown in Table 1. Specific clinical characteristics of the two in-house cohorts are shown in Table 2.

### SSD classification based on normative deviations

A RF classifier was trained using leave-site-out CV, producing the results summarized in Table 3. We additionally performed 1,000 random permutations to estimate mean performance values across folds. Notably, all observed performance metrics lay outside the 95% confidence interval of the permutation-based distribution. For our 10-fold CV-results across the entire cohort we had a mean performance of: Accuracy = 70.7%, Balanced Accuracy = 69.42%, F1-Score = 0.64, Precision = 66.66%, ROC-AUC = 0.76.

**Table 3:** Performance Metrics of the Random Forest Classifier Using Leave-Site-Out Cross-Validation.

Left-Out Site	Accuracy	Balanced Accuracy	F1-Score	Precision	ROC-AUC
NSS_BMBF	65.03%	64.43%	0.54	48.61%	0.66
whiteCAT-URBN	64.63%	64.46%	0.53	78.37%	0.73
UCLA	63.33%	63.33%	0.58	68.18%	0.69
Cobre	68.71%	68.76%	0.66	72.58%	0.68
Mean-Performance	65.43%	65.25%	0.58	66.93%	0.69
Permutation-CI 95%	46.39–54.67%	45.8–54.08%	0.35–0.45	39.89–51.64%	0.45–0.55

This table presents the classification performance of the Random Forest (RF) model, trained using a leave-site-out cross-validation (CV) approach. It includes key evaluation metrics for each left-out site, offering insights into the model’s generalizability and its effectiveness in identifying structural deviations in SSD across independent datasets while minimizing site-specific biases. The 95% confidence intervals (CI) were derived through permutation testing, were labels were randomly shuffled.

### Ranking feature importance for SSD classification with mean decrease in impurity

**Fig. 1:**
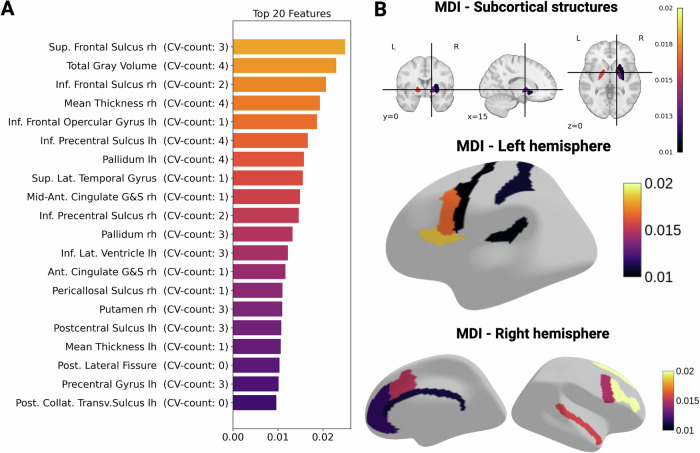
Key Brain Regions Contributing to the Classification of SSD vs. HC based on normative deviations. This figure displays the feature importance (mean decrease in impurity) for key brain regions used to classify individuals with SSD and HC. **A** The y-axis represents the brain regions identified by the classifier, while the x-axis indicates the feature importance, with higher values reflecting greater contribution to the classification. CV-counts reflects the number of times the feature was in the top 20 highest MDI-values during the leave-one-site-out CV. **B** An illustration of where these key brain regions are located in the brain.

For the calculation of the MDI, the RF model was trained on data from all four inhouse datasets and two publicly available datasets. Figure 1 illustrates the highest 20 MDI-values. Notably, total gray matter volume (GMV) emerged as the feature that is simultaneously important in all four cohorts, and has the second highest MDI.

### Association between brain deviations and cognition, neurological soft signs, and psychopathology

**Fig. 2:**
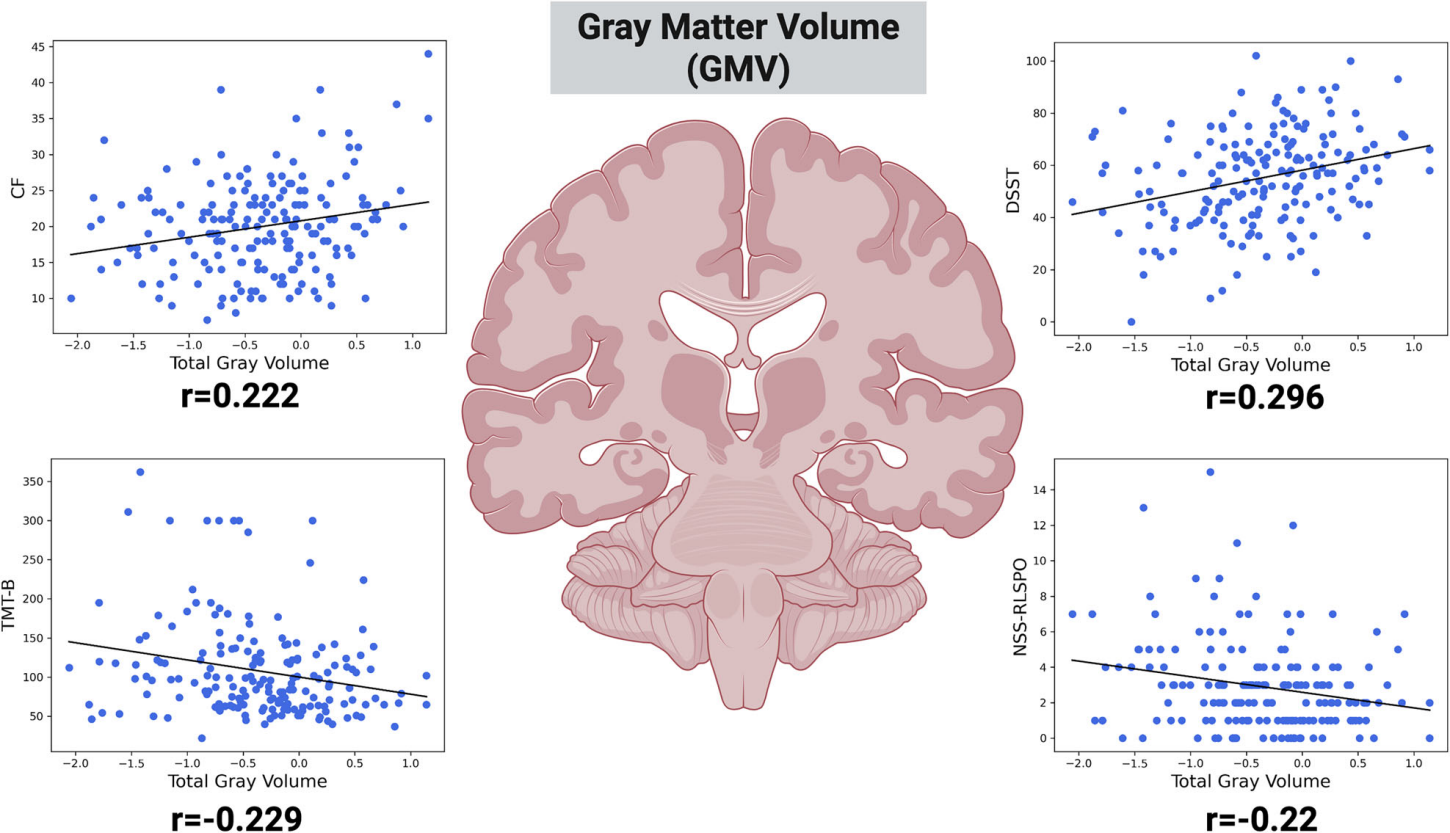
Associations of gray matter volume (GMV) with cognitive and sensorimotor measures. Scatter plots show Pearson’s correlations (after FDR-correction) between total GMV (x-axis) and four clinical indices (y-axis): CF (top left, r = 0.222); DSST (top right, r = 0.296); TMT-B (bottom left, r = –0.229); and NSS-RLSPO (bottom right, r = –0.220). Each point represents an individual participant, and the solid line denotes the best linear fit. Abbreviations: CF: Categorical Fluency; DSST: Digit Symbol Substitution Test; TMT-B: Trail-Making-Test B; NSS-RLSPO: Neurological Soft Signs Subscale Right/Left and Spatial Orientation.

**Fig. 3:**
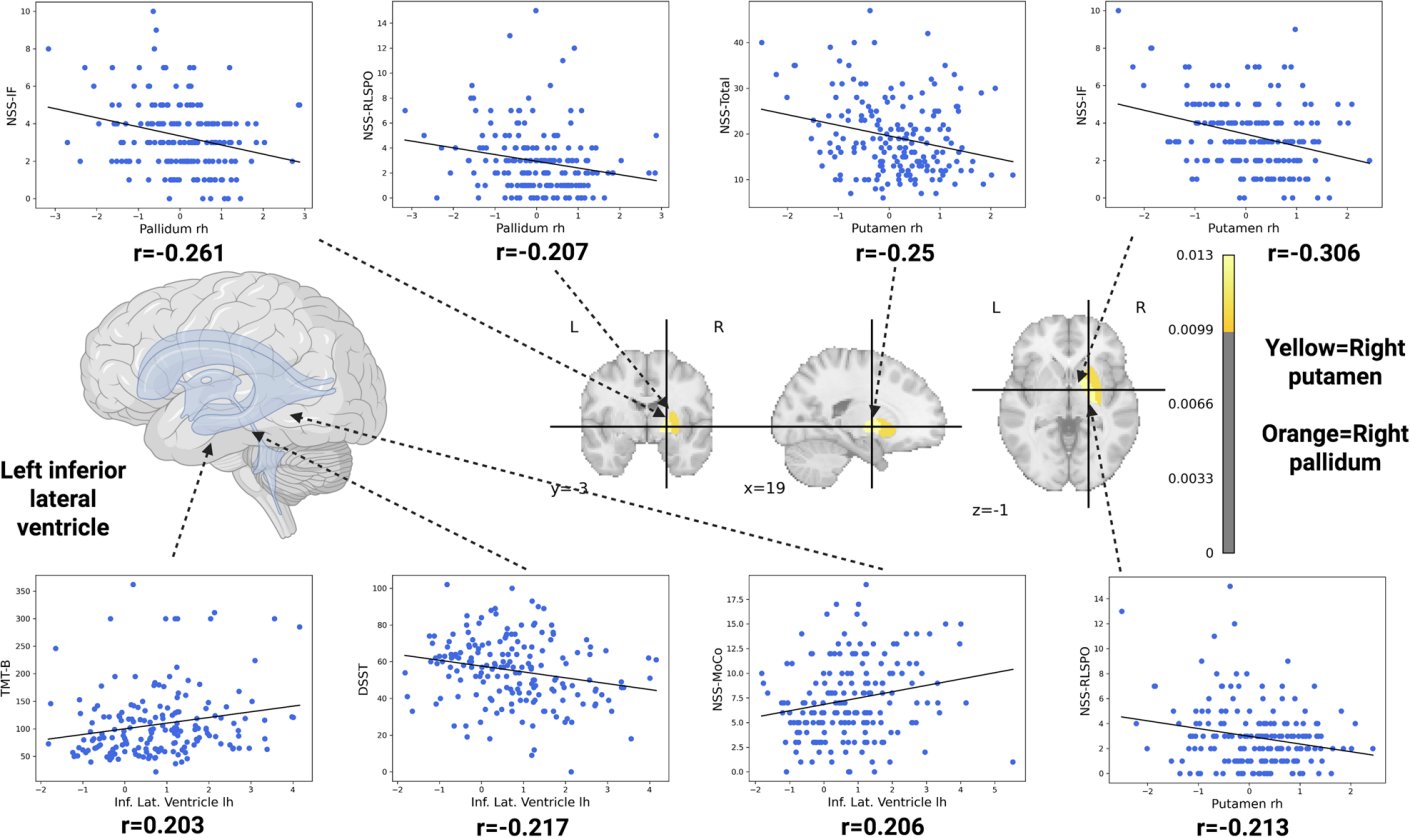
Associations of right pallidum and putamen and left inferior lateral ventricle morphology with cognitive and sensorimotor measures. The scatter plots (top row) show negative Pearson’s correlations (after FDR-correction) between right pallidum/putamen morphology and sensorimotor measures (NSS-IF, NSS-RLSPO, NSS-Total), with correlation coefficients (*r*) ranging from –0.2 to –0.306. The scatter plots (bottom row) illustrate Pearson’s correlations (after FDR-correction) between left inferior lateral ventricle morphology and right putamen and cognitive tasks (TMT-B and DSST) as well as sensorimotor performance (NSS-MOCO and NSS-RLSPO), with *r* values ranging from –0.2 to 0.217. In each plot, points represent individual participants, and the regression line depicts the direction and strength of the association.

**Fig. 4:**
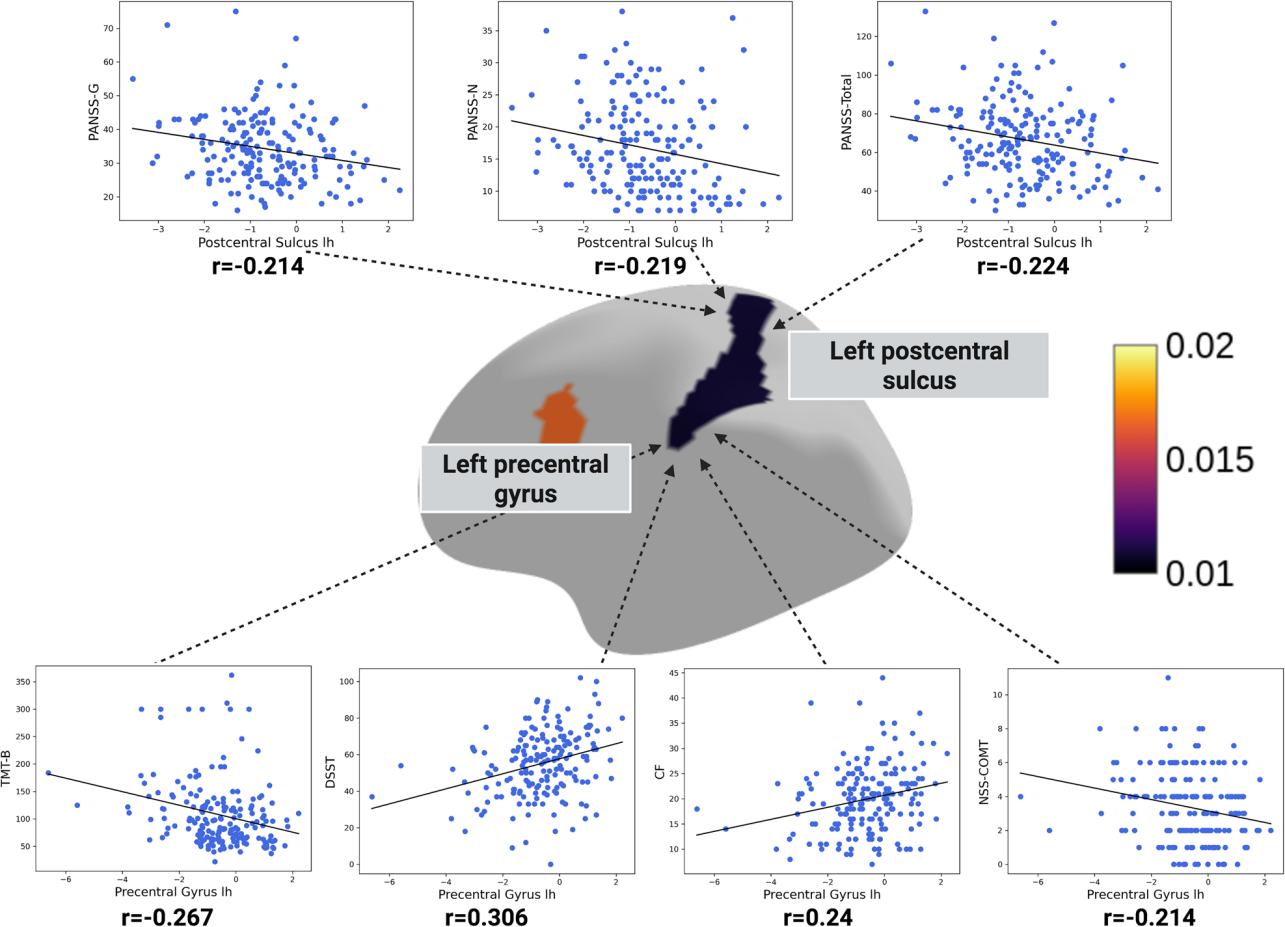
Associations of left postcentral and left pericallosal sulcal morphology with psychopathological, cognitive and sensorimotor measures. The scatter plots (top row) show negative Pearson’s correlation after FDR-correction between left postcentral sulcus and psychopathological symptoms (PANSS-G, PANSS-N, and PANSS-Total), with correlation coefficients (*r*) ranging from −0.214 to −0.224. The scatter plots (bottom row) illustrate Pearson’s correlation after FDR-correction between left pericallosal sulcus and performance on cognitive/sensorimotor tests, including TMT-B (r = −0.267), DSST (r = 0.306), verbal/category fluency (CF; r = 0.24), and motor coordination (NSS-MOCO; r = −0.214). In each plot, blue dots represent individual participants, and the regression line depicts the direction and strength of the association.

In our univariate testing, we constructed correlation matrix of the previously selected brain features and cognition, NSS and psychopathology. Total GMV was significantly correlated with the trail-making test (TMT-B) (r = −0.23, q = 0.03), digit-symbol-substitution-test (DSST) (r = 0.3, q = 0.003), categorical fluency (CF) (r = 0.22, q = 0.04), and NSS RLSPO (r = −0.23, q = 0.03) scores (Fig. 2). The right pallidum correlated with NSS IF (r = −0.26, q = 0.001) and RLSPO (r = −0.21, q = 0.04) scores (Fig. 3). Left lateral inferior ventricle correlated with worse DSST (r = −0.22, q = 0.04), TMT-B (r = 0.2, q = 0.05), and NSS MOCO (r = 0.21, q = 0.04) scores (Fig. 3). The volume of the right putamen correlated negatively with NSS total (r = −0.25, q = 0.02), IF (r = −0.31, q = 0.002) and RLSPO (r = −0.21, q = 0.04) scores (Fig. 3). Further, the left postcentral sulcus correlated negatively with negative (r = −0.21, q = 0.04), general (r = −0.22, q = 0.04) and total (r = −0.22, q = 0.04) scores of the PANSS (Fig. 4). Lastly, the thickness of the left precentral gyrus correlated negatively with the TMT-B (r = −0.28, q = 0.01) and NSS COMT (r = −0.21, q = 0.04) scores, and positively with DSST (r = 0.31, q = 0.002) and CF (r = 0.24, q = 0.02) scores (Fig. 4). Across the FDR-significant associations, Pearson’s r and Spearman’s ρ were generally similar in direction and magnitude, and leave-one-out analyses showed a relatively narrow range of Pearson’s r estimates (e.g., Total Gray Matter–TMT-B: r = −0.23; LOO range −0.25 to −0.20; Precentral Gyrus lh–DSST: r = 0.31; LOO range 0.29 to 0.32). Re-estimating Pearson’s r after excluding potentially influential observations resulted in small changes for several pairs (e.g., Total Gray Matter–TMT-B: r excluded = −0.24; Precentral Gyrus lh–DSST: r excluded = 0.33; Putamen rh–NSS-IF: r = −0.31, r excluded = −0.28), whereas larger changes were observed for others (e.g., Postcentral Sulcus lh–PANSS-G: r = −0.22 vs r_excluded = −0.09; Postcentral Sulcus lh–PANSS-Total: r = −0.22 vs r_excluded = −0.12; Putamen rh–NSS-Total: r = −0.25 vs r_excluded = −0.15). Full results are provided in Table 4.

**Table 4:** Sensitivity analysis of all FDR-significant Brain-Deviation-Clinical pairs.

Brain Deviation	Clinical variable	Pearson r	p	Spearman rho	p	Pearson r min	Pearson r max	Potential influential observations n	Pearson r excluded	p excluded
Total Gray Matter	TMT-B	−0.23	1.90e-03	−0.23	1.95e-03	−0.25	−0.20	25	−0.24	2.55e-03
Total Gray Matter	NSS-RLSPO	−0.22	2.59e-03	−0.21	3.64e-03	−0.24	−0.19	24	−0.25	1.55e-03
Precentral Gyrus lh	TMT-B	−0.27	2.62e-04	−0.30	2.88e-05	−0.29	−0.24	24	−0.28	4.10e-04
Precentral Gyrus lh	DSST	0.31	2.73e-05	0.31	2.30e-05	0.29	0.32	23	0.33	2.59e-05
Postcentral Sulcus lh	PANSS-N	−0.22	3.39e-03	−0.25	7.52e-04	−0.25	−0.19	22	−0.17	2.90e-02
Postcentral Sulcus lh	PANSS-G	−0.21	2.77e-03	−0.19	8.42e-03	−0.24	−0.19	21	−0.09	2.59e-01
Postcentral Sulcus lh	PANSS-Total	−0.22	2.17e-03	−0.21	4.80e-03	−0.25	−0.19	21	−0.12	1.41e-01
Putamen rh	NSS-Total	−0.25	6.07e-04	−0.19	8.25e-03	−0.27	−0.22	21	−0.15	6.34e-02
Putamen rh	NSS-IF	−0.31	2.30e-05	−0.25	6.94e-04	−0.33	−0.26	21	−0.28	2.76e-04
Total Gray Matter	DSST	0.30	5.11e-05	0.30	4.48e-05	0.28	0.32	20	0.30	1.29e-04
Inferior Lateral Vetntricle lh	TMT-B	0.20	5.92e-03	0.22	2.51e-03	0.17	0.23	20	0.17	2.92e-02
Putamen rh	NSS-RLSPO	−0.21	3.66e-03	−0.14	6.21e-02	−0.23	−0.16	20	−0.13	8.71e-02
Left Precentral Gyrus	CF	0.24	1.05e-03	0.26	4.37e-04	0.22	0.27	19	0.23	3.49e-03
Pallidum rh	NSS-RLSPO	−0.21	4.60e-03	−0.24	1.03e-03	−0.23	−0.19	19	−0.22	3.93e-03
Total Gray Matter	CF	0.22	2.57e-03	0.17	2.45e-02	0.18	0.25	18	0.16	3.59e-02
Left Precentral Gyrus	NSS-COMT	−0.21	3.37e-03	−0.23	1.97e-03	−0.24	−0.19	18	−0.19	1.16e-02
Inferior Lateral Vetntricle lh	NSS-MOCO	0.21	4.90e-03	0.23	1.78e-03	0.18	0.25	17	0.24	1.46e-03
Inferior Lateral Vetntricle lh	DSST	−0.22	3.37e-03	−0.24	1.06e-03	−0.23	−0.20	17	−0.24	2.26e-03
Pallidum rh	NSS-IF	−0.26	3.41e-04	−0.26	3.81e-04	−0.28	−0.23	17	-0.25	1.19e-03

Analysis included additional Spearman correlation, recomputing Pearson correlations with leave-one-subject-out (Pearson r min/max), and recomputing Pearson correlation without subject’s that are potential outliers identified with Cook’s D.

*lh* left hemisphere, *rh* right hemisphere, *PANSS* Positive and Negative Syndrome Scale; *N* Negative Symptoms Subscale, *G* General Symptoms Subscale, *NSS* Neurological Soft Signs, *RLSPO* Right/Left and Spatial Orientation, *IF* Integrative Function, *COMT* Complex Motor Tasks, *MOCO* Motor Coordination, *DSST* Digit Symbol Substitution Test, *TMT-B* Trail Making Test B, *CF* Categorical Fluency.

**Fig. 5:**
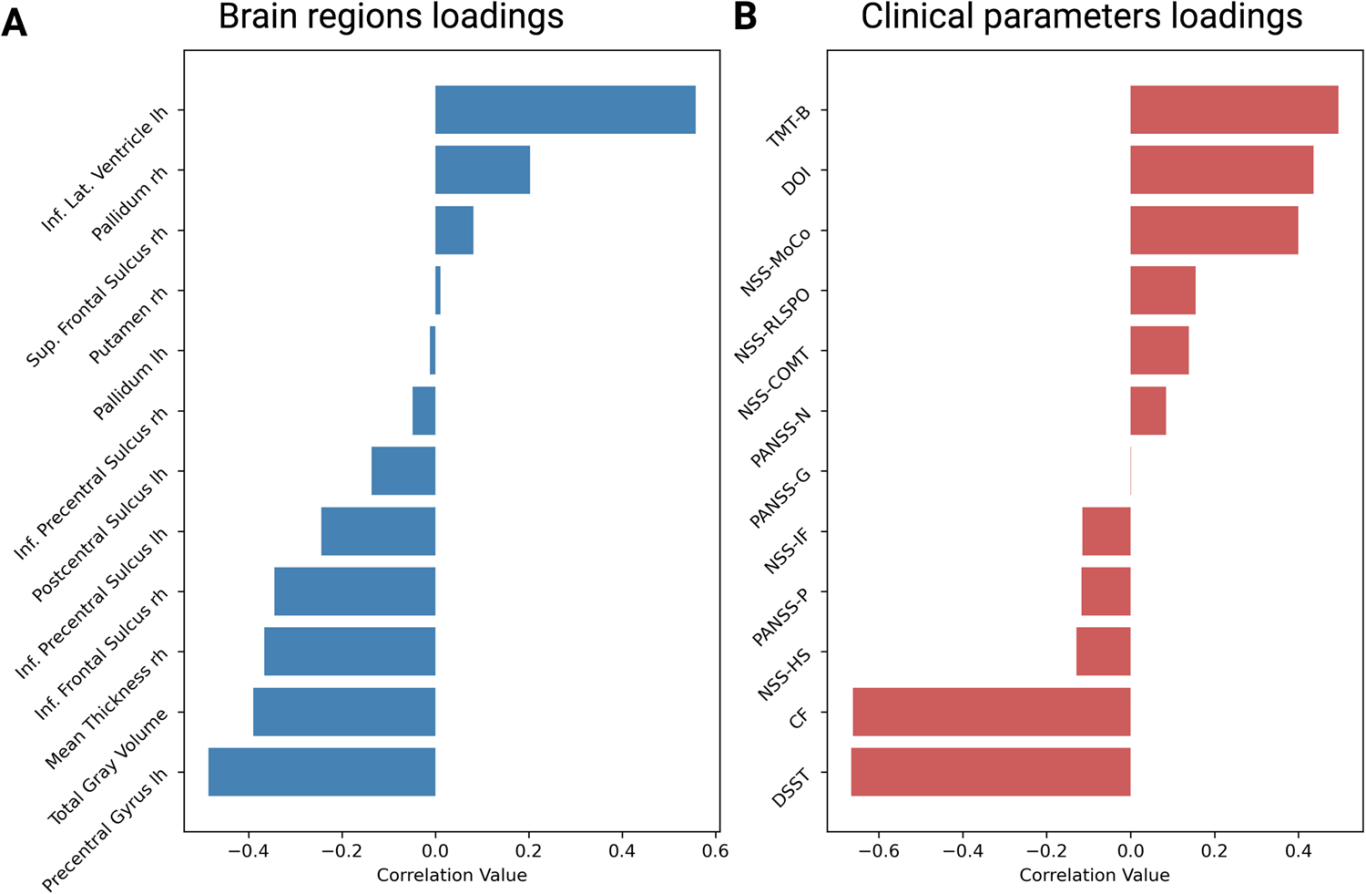
Canonical correlation analysis linking brain deviations and clinical parameters. Correlation values between (**A**) brain regions deviations and (**B**) clinical parameters together with their respective loading of the first canonical mode. *Abbreviations:* lh: left hemisphere; rh: right hemisphere; TMT-B: Trail Making Test B; NSS: Neurological Soft Signs scale; NSS-MoCo: NSS Subscale Motor Coordination; DOI: Duration of illness; NSS-RLSPO: NSS Subscale Right/Left and Spatial Orientation; COMT: NSS Subscale Complex Motor Tasks; PANSS: Positive and Negative Syndrome Scale; PANSS-G: PANSS General Symptoms Score; PANSS-N: PANSS Negative Symptoms Score; PANSS-P: PANSS Positive Symptoms Score; NSS-HS: NSS Subscale Hard Signs; NSS-IF: NSS Subscale Integrative Functioning; DSST: Digit Symbol Substitution Test; CF: Categorical Fluency.

In our final analysis, we explored the relationship between psychopathology, sensorimotor alterations, and cognitive functioning and brain variations by examining the first canonical variate derived from the CCA, based on the SSD patients in the whiteCAT and NSS cohorts. Permutation testing, conducted with 1,000 random permutations, revealed a significant difference between the observed canonical correlation values (r = 0.56, p = 0.002) and the permuted distribution (Fig. 5). The findings indicate that a longer duration of illness, higher NSS MOCO scores, and lower cognitive scores were associated with increased volumes of the left inferior lateral ventricle and right pallidum, along with reduced mean CT and GMV in the right hemisphere, and reduced thickness in the precentral gyrus in the left hemisphere.

**Fig. 6:**
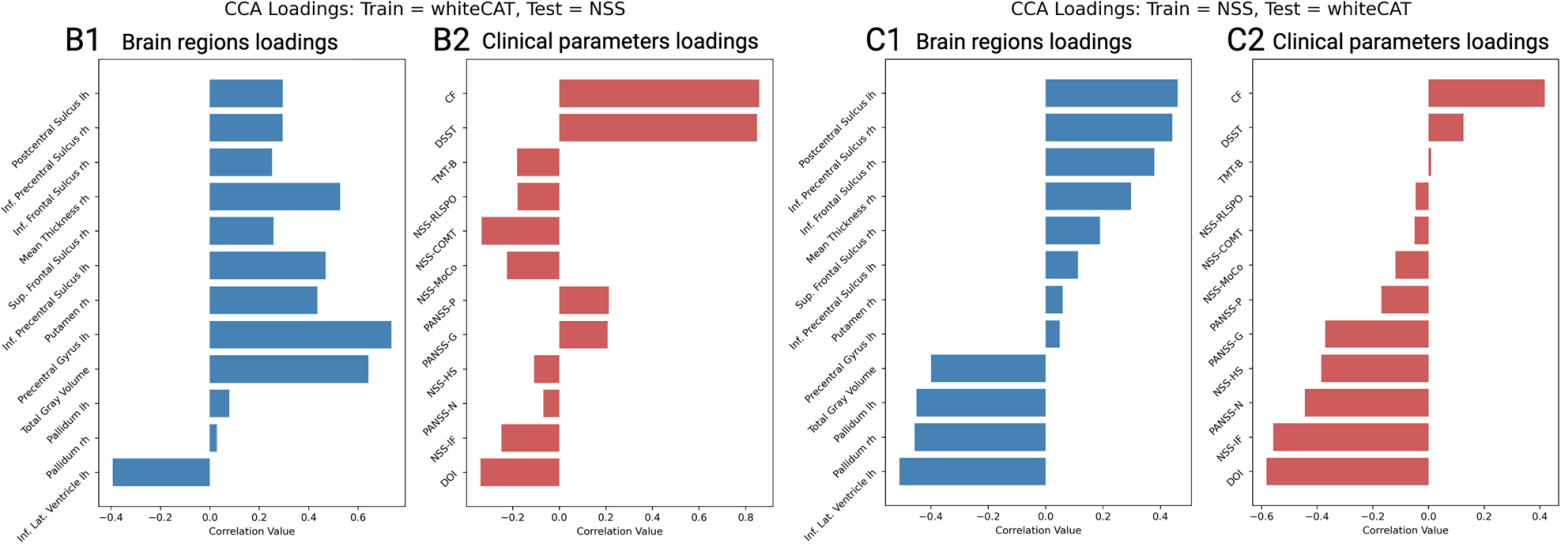
Out-of-site validation of the canonical correlation analysis (CCA). The figure illustrates the correlation of individual variables with their respective canonical scores (i.e., canonical loadings), highlighting the underlying correlation structure and indicating which variables contributed most robustly to the validated canonical solution. *Abbreviations:* lh: left hemisphere; rh: right hemisphere; TMT-B: Trail Making Test B; NSS: Neurological Soft Signs scale; NSS-MoCo: NSS Subscale Motor Coordination; DOI: Duration of illness; NSS-RLSPO: NSS Subscale Right/Left and Spatial Orientation; COMT: NSS Subscale Complex Motor Tasks; PANSS: Positive and Negative Syndrome Scale; PANSS-G: PANSS General Symptoms Score; PANSS-N: PANSS Negative Symptoms Score; PANSS-P: PANSS Positive Symptoms Score; NSS-HS: NSS Subscale Hard Signs; NSS-IF: NSS Subscale Integrative Functioning; DSST: Digit Symbol Substitution Test; CF: Categorical Fluency.

For completeness, out-of-site validation of the CCA showed that the model derived from the whiteCAT cohort could be validated on the NSS cohort (*r* = 0.35, *p* < 0.01), whereas the model derived from the NSS cohort could not be validated on the whiteCAT cohort (*r* = 0.15, *p* = 0.21). Figure 6 provides a detailed overview of these findings, illustrating how each variable correlates with its respective canonical scores (i.e., canonical loadings), showing a similar but inverted loadings in the CCA trained on the whiteCAT cohort.

### Extreme value analysis

**Fig. 7:**
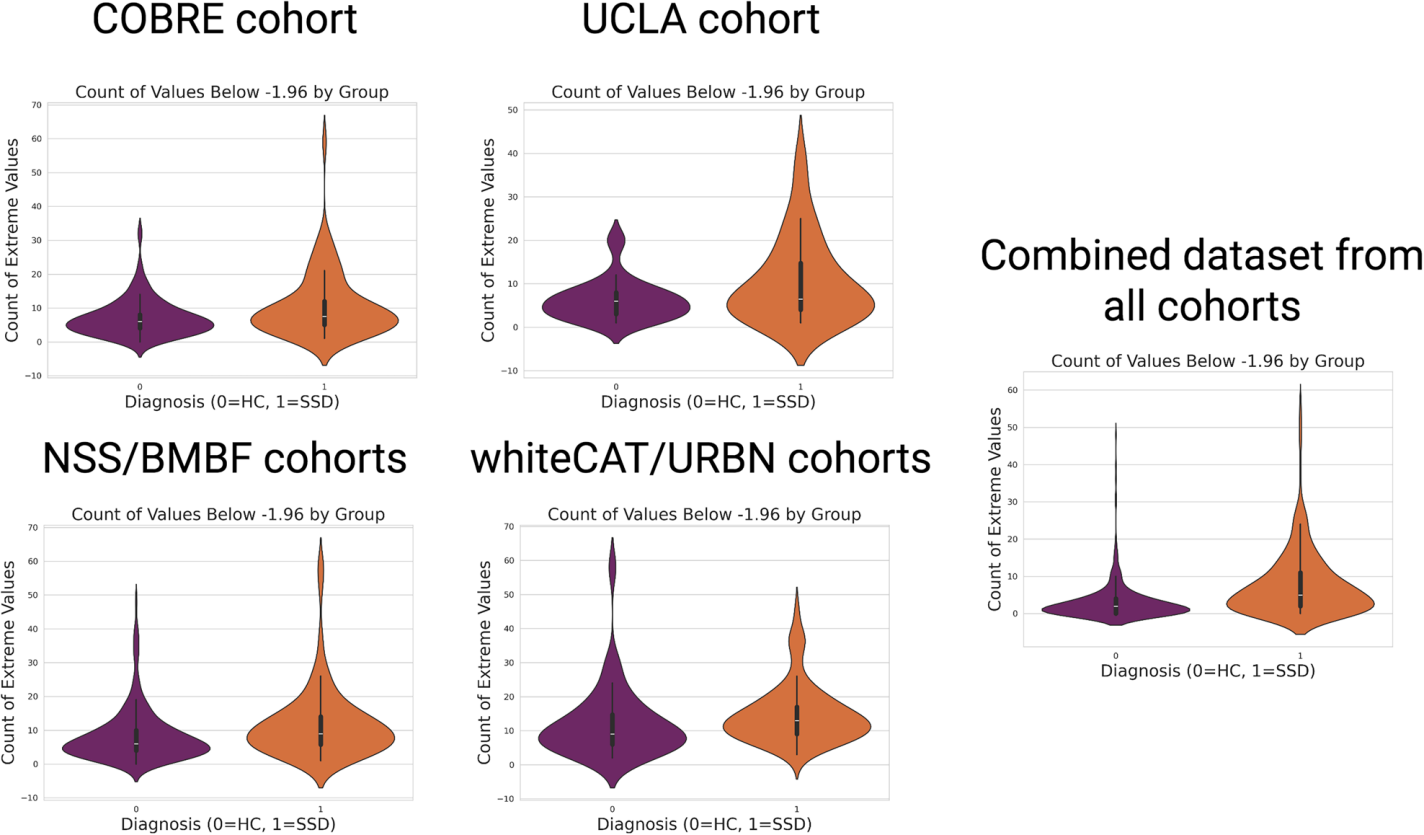
Violin plots illustrating the distribution of extreme values, separated by site and diagnosis. The plots show the count of extreme values for each diagnostic group (e.g., schizophrenia, healthy controls) across different sites. The shape and spread of the violins represent the distribution of extreme values, providing insights into the variability and potential site-related differences in the data.

To characterize the heterogeneity of normative deviations in individuals with SSD, we quantified the total count of extreme values across all brain regions. Extreme values were defined as those falling outside the range of ±1.96 standard deviations from the mean (for overview across all cohorts see Fig. 7). For this analysis we focus on extreme negative deviations, i.e., values smaller than −1.96. We have a significantly lower count of extreme deviations in the HC group (β = 0.06, SE = 0.01, 95% CI [0.04, 0.08] p = 3.76 × 10⁻^8^), calculated via GEE with a binomial distribution, site as grouping effect and age and sex as covariates. Across all cohorts, 265 of 309 (85.58%) SSD patients had at least one region that showed and negative extreme deviation while this was the case for 346 of 502 (69.92%) HC that had an extreme negative deviation. If we look closer into the extreme deviations of SSD patients we can see that the representation of extreme deviations is highly heterogeneous with only 5 brain features of 177 have extreme negative deviations in more than 10% of the patients, where the precentral gyrus of the right hemisphere (13.79%), the right inferior precentral sulcus (13.10%), right superior frontal sulcus (12.41%), left hippocampus (12.07%), and left occipital-temporal medial lingual gyrus (10.34%).

To investigate the associations between extreme brain deviations and psychopathology, cognitive functioning, and sensorimotor alterations we analyzed a subset of participants for whom complete behavioral data were available (N = 184), collected from the whiteCAT and NSS cohort. In our initial analysis, we employed a linear mixed model with age and sex as covariates to assess the relationship between the number of extreme counts per subject and DOI, PANSS, TMT-B, DSST, CF, and NSS scores. After applying FDR correction, we identified significant associations with the DOI (β = 0.22, SE = 0.09, 95% CI [0.05, 0.4], q = 2.13 × 10⁻^2^), TMT-B (β = 0.05, SE = 0.01, 95% CI [0.03, 0.07], q = 3.29 × 10⁻^4^), DSST (β = −0.1, SE = 0.03, 95% CI -[0.16, −0.05], q = 1.18 × 10⁻³), and CF (β = −0.19, SE = 0.06, 95% CI -[0.32, −0.07], q = 5.0. × 10⁻³). Significant relationships were also observed with NSS total score (β = 0.36, SE = 0.09, 95% CI [0.19, 0.53], q = 3.05 × 10⁻^4^), NSS MOCO (β = 0.66, SE = 0.18, 95% CI [0.31, 1.02], q = 9.21 × 10⁻⁴), NSS IF (β = 1.4, SE = 0.37, 95% CI [0.63, 2.1], p = 9.21 × 10⁻⁴), NSS COMT (β = 0.89, SE = 0.35, 95% CI [0.21, 1.57], q = 2.04 × 10⁻²), and NSS RLSPO (β = 0.7, SE = 0.28, 95% CI [0.14, 1.25], q = 2.13 × 10⁻²). However, no significant associations were observed with the PANSS total score or its subscales.

## Discussion

In this multi-dataset study, we used NM to quantify individual deviations from a reference brain profile and evaluated whether these deviation signatures (i) support clinically relevant classification of SSD and (ii) map onto symptom-relevant phenotypes, including psychopathology, cognition, and NSS. Importantly, we designed the analysis to reflect a realistic clinical deployment scenario: models were trained/fine-tuned on a subset of HC and then applied to SSD patients, and leave-site-out CV directly tested whether deviation-based inference generalizes across clinics rather than overfitting site-specific idiosyncrasies. This framing moves NM beyond a methodological exercise by explicitly interrogating its practical utility for cross-site biomarker development.

This study yielded three main findings: First, using a NM approach (i.e., deviation scores), we achieved a mean balanced accuracy of 65% in predicting SSD. These features performed comparably to other SSD predictive models, and their robustness underscores the usefulness of tools like the PCNtoolkit in mitigating cross-site and cross-study variability. Feature importance analysis (MDI) revealed weak but consistent markers (e.g., GMV), with bootstrapping and leave-site-out CV providing additional reliability. Second, in-depth analyses of our deeply phenotyped cohorts (whiteCAT and NSS) and canonical correlation analysis (CCA) uncovered distinct links between these brain deviations and clinical parameters, including psychopathology, cognition, and NSS. Third, consistent with previous research, we observed considerable heterogeneity in normative brain deviations across cohorts. Only five features were consistently identified as showing extreme deviations in more than 10% of SSD patients, with the right precentral sulcus (M1) emerging as the most frequently deviating region (13.79%). Furthermore, a higher count of extreme negative deviations correlated with poorer cognitive performance and elevated NSS scores, although no significant association emerged with PANSS scores.

The first finding is not surprising, because this level of accuracy, attained through leave-site-out CV, is comparable to results from smaller-scale studies [51, 52]. Our findings indicate that SSD can be weakly but reliably predicted across four independent cohorts. Despite low levels of MDI across sites, the right superior frontal sulcus and total GMV emerged as the most predictive features, underscoring its importance in the classification of SSD. However, the patterns of brain deviations varied substantially within and across sites, suggesting that neuroanatomical alterations in SSD are not uniform. In previous MRI studies, changes of GMV [53] and CT [8] as well as volumetric alterations of the superior frontal areas [54], lateral ventricles [55], precentral gyrus [56], pallidum [57], postcentral sulcus [58], and putamen [59] were assumed to be important neuronal correlates of the SSD. However, weaker classification accuracy of 70% over the entire cohort than previous NM classification approaches show a possible greater heterogeneity in this cohort [23]. Overall, the toolkit’s adaptability to site-specific data underscores its promise for future data integration efforts.

Second, univariate analyses revealed that higher total NSS scores were associated with reduced sensorimotor regions such as basal ganglia and precentral gyrus. Specifically, the NSS total score and IF and RLSPO subscale scores correlated with right pallidum and putamen volumes. NSS subscale scores MOCO and COMT were associated with larger volumes of the left lateral inferior ventricle and lower volumes of the precentral gyrus. These observations are consistent with previous findings demonstrating a relationship between NSS severity and volumetric alterations in sensorimotor regions [60]. Furthermore, the dilation of the lateral ventricles appears to result from decreased basal ganglia volume, a finding consistently reported in previous studies [61–63]. From a pathophysiological perspective, the precentral gyrus encompasses the primary motor cortex (M1), a critical hub for voluntary movement and the origin of key sensorimotor pathways such as the corticospinal tract [64]. Structural and functional alterations in M1 may impair coordinated and purposeful movements in SSD patients, which is reflected by elevated NSS scores. Taken together, the identified associations between NSS and both the pallidum and putamen as well as M1 deviations reinforce the notion that NSS are markers of structural changes in the sensorimotor system and might reflect both aberrant brain neurodevelopment and neurodegeneration [61–63]. All but the association of the right putamen and NSS total score remained robust in our sensitivity analysis. Here, the correlation was stable in our Spearman and LOO-Pearson correlation analysis, however, when excluding potential influential observations identified by Cook’s D the r-value decreased from −0.25 to −0.15, highlighting a potentially higher sensitivity to certain observations, potentially reflecting greater heterogeneity, non-linearity, or measurement noise in these clinical measures.

A very recent study by Huang et al. [27] utilized NM based on the Human Connectome Project Lifespan datasets to characterize typical developmental trajectories of thalamic nuclei volumes and applied these models to an independent clinical cohort of SSD individuals. This study showed that up to 18% of SSD patients exhibited abnormally small mediodorsal and pulvinar thalamic volumes, and the extent of these deviations, rather than raw volumes, correlated with the severity of cognitive impairment. Notably, our findings are in accordance with previous studies [65], as we also observed that cognitive impairments in terms of higher TMT-B, DSST and CF scores in SSD are linked to alterations in the left inferior lateral ventricle and the left precentral gyrus. These associations remained robust under different analysis variations, shown in our sensitivity analysis, highlighting a potentially robust link for future research towards biomarker discovery.

CT alterations in the postcentral sulcus were associated with higher PANSS negative, general and total scores, suggesting a potential link between somatosensory cortical morphology and SSD symptom burden. However, these relationships appeared sensitive to influential observations, most notably for PANSS general and total, so they should be interpreted cautiously and prioritized for replication in independent samples. This caution is consistent with prior cortical-thickness work reporting null symptom–structure relationships in closely related sensorimotor territories: for example, in antipsychotic-naïve first-episode schizophrenia, CT in the right postcentral gyrus was not correlated with PANSS (or global functioning), despite case–control differences in this region, and subgroup analyses likewise did not differentiate patients with versus without prominent negative symptoms [66]. More broadly, Oertel-Knöchel et al. reported no significant correlations between any PANSS scores and cortical thickness across their assessed regions, underscoring heterogeneity in CT–PANSS coupling across samples and analytic choices [67]. Nevertheless, the postcentral gyrus encompasses the primary somatosensory cortex, central to proprioception and the integration of tactile, pressure, temperature and pain-related inputs [68], and structural and functional alterations in this system have increasingly been implicated in SSD, highlighting the sensorimotor network as a candidate locus of pathophysiological change [69]. Emerging neuroimaging evidence further supports the involvement of the postcentral region and broader sensorimotor circuits in both early and chronic stages of SSD. Zhao et al. [70] demonstrated that functional connectivity disruptions in first-episode and chronic SSD converge in the pre- and postcentral cortices, suggesting that these regions represent common pathological nodes across illness stages. Notably, in early-stage SSD, abnormal connectivity is more localized to areas involved in mouth movement, while in later stages, it extends to broader sensorimotor territories, pointing to a potential progression in network involvement. Similarly, Ferro et al. [58] reported reduced right postcentral gyrus volume in first-episode SSD patients, particularly in males, underscoring early structural vulnerability in this region. Taken together, these findings support the relevance of postcentral/sensorimotor abnormalities in SSD while also indicating that CT–symptom associations, especially those involving broad PANSS composites, may be modest and sample-sensitive. More generally, the limited associations observed with PANSS scores contrasted with robust relationships between normative brain deviations, cognition, and sensorimotor measures suggest that a substantial portion of structural brain alterations in SSD may be more closely tied to enduring cognitive and sensorimotor dysfunction than to current psychopathology. This interpretation is consistent with mounting evidence that cognitive deficits and sensorimotor abnormalities represent core, trait-like features of SSD [71–73], whereas symptom dimensions captured by instruments such as the PANSS are often more state-dependent, fluctuating with illness phase and treatment response.

The multivariate analysis revealed a robust first canonical mode, validated through permutation testing, which demonstrated a significant linkage between brain and behavioral features in SSD. However, the leave-site out CV showed these correlations can be validated when trained on the whiteCAT cohort, but not when trained on the NSS cohort (s. Figure 6). This highlights the exploratory nature of this finding and the need for larger deeply phenotyped cohorts. On one side, positive correlations were observed with structural loadings in the inferior lateral ventricle and pallidum, alongside behavioral measures such as TMT-B, NSS MOCO, and DOI. On the other side, negative correlations were found between GMV, right mean CT, M1 and cognitive tests like the DSST and CF. These findings underscore the central role of cognitive impairment in SSD, highlighting its close association with both structural brain alterations and sensorimotor abnormalities, particularly motor coordination deficits as indexed by NSS. Rather than suggesting a unidirectional relationship, our data support a dynamic, bidirectional interaction between cognitive and sensorimotor systems [74]. This aligns with developmental theories, such as the Dynamic Systems Approach by Thelen and Smith [75], which conceptualize cognition as emerging from continuous interactions between motor behavior, perception, and environmental engagement. In this framework, sensorimotor processes are foundational to cognitive development and remain tightly integrated across the lifespan. In SSD, the co-occurrence of cognitive and sensorimotor dysfunction may reflect shared neurodevelopmental disturbances in brain circuits subserving both domains. Specifically, structural deviations such as ventricular enlargement may index broad disruptions in systems relevant to coordinated movement, executive function, and goal-directed behavior. Recognizing the interdependence of cognition and sensorimotor function offers a more mechanistic understanding of SSD and suggests new avenues for early detection and intervention.

Specifically, the observed associations between brain ventricle volume and both cognitive [76] and sensorimotor [77] performance align with previous findings in the field [78]. Recent evidence indicates that sensori-/psychomotor dysfunction is not merely an epiphenomenon of medication or acute illness, but a stable, trait-like feature of SSD, emerging early in development and persisting across the illness course [79, 80]. Structural deviations such as ventricular enlargement may thus reflect broader neurodevelopmental disturbances in brain systems supporting both cognition and sensori-/psychomotor function [81]. Further, these insights reinforce growing calls to expand our conceptualization of SSD beyond the traditional emphasis on positive and negative symptoms [82]. Historically central but later marginalized, sensori-/psychomotor abnormalities ranging from catatonia and dyskinesia to NSS and gesture impairments are increasingly recognized as core, transdiagnostic features with distinct neural correlates across motor, premotor, and cerebello-thalamo-cortical circuits [73]. Clinically, these sensori-/psychomotor phenomena are highly prevalent [80], measurable with high reliability, and often present early, making them valuable markers for diagnosis, staging, and prediction of functional outcomes [83, 84]. Furthermore, sensori-/psychomotor markers hold significant promise for informing individualized treatment strategies, including early intervention, rehabilitation, and neuromodulation targeting sensori-/psychomotor-related brain circuits [85]. To advance this translational potential, future clinical trials should incorporate sensorimotor parameters derived from MRI studies as primary or secondary outcome measures. The inclusion of a sensori-/psychomotor abnormalities in emerging nosological frameworks such as RDoC [86] and HiTOP [87] further underscores that these features are not peripheral, but fundamental to understanding serious mental illness. Finally, recognizing sensori-/psychomotor dysfunction alongside cognitive and affective domains not only enhances diagnostic and prognostic precision but may also help shape a more comprehensive reconceptualization and eventual renaming of SSD [88]. The significant role of DOI further suggests that these cognitive and sensori-/psychomotor impairments may manifest progressively, pointing to potential long-term consequences of a chronic or evolving illness trajectory in SSD. This integrated neurobiological perspective highlights the importance of considering both cognitive and sensori-/psychomotor alterations when exploring the pathophysiology of SSD, offering valuable insights for future diagnostic and therapeutic approaches.

As the study’s third finding, further analysis revealed substantial heterogeneity in normative deviations across cohorts. However, only five brain features were consistently identified as extremely different in more than 10% of SSD patients, with the right precentral gyrus emerging as the most frequent (13.79%). On average, SSD patients exhibited more extreme deviations than HC across all sites, emphasizing the broader neuroanatomical disruption in SSD. This heterogeneity was previously identified in previous smaller studies of NM [2]. For instance, Wolfers et al. [2] found an overlap of normative deviations in only 2% of the patients and Jinglei et al. [28] also showed heterogeneous structural alterations, where only 79% showed extreme deviations in at least one brain region. Additionally, in our extensively phenotyped in-house cohorts, the relationship between extreme brain deviations and behavioral parameters offered valuable insights into the brain-behavior relationship. Specifically, a higher number of extreme deviations was linked to poorer cognitive performance and elevated NSS scores, indicating that age-dependent global negative brain deviations are associated with distinct clinical parameters. Overall, these findings suggest that specific brain deviations may be linked to functional outcomes in SSD, particularly in cognitive and sensorimotor domains. Features with high predictive value for SSD classification (top 20 based on MDI and CV-count >= 2) further elucidated these relationships. The marked heterogeneity of extreme deviations observed in our study is consistent with a growing body of neuroimaging evidence indicating that SSD are characterized by substantial interindividual variability in brain structure, extending beyond group-level mean differences. Early meta-analytic work by Brugger et al. [89] demonstrated that patients with first-episode SSD exhibit significantly greater variability in regional brain volumes, particularly in the putamen, thalamus, temporal cortex, and ventricles while showing reduced variability in the anterior cingulate cortex, suggesting a combination of heterogeneous and core neurobiological features within SSD. This concept was further substantiated by Alnæs et al. [90], who showed that SSD are associated with increased dispersion in cortical thickness, cortical area, ventricular volumes, and hippocampal subfields across large multi-site samples. Importantly, while polygenic risk for SSD was associated with thinner frontotemporal cortices and smaller hippocampal subfield volumes, it was *not* associated with increased heterogeneity. This dissociation suggests that structural variability in SSD may arise from complex gene–environment interactions, illness-related processes, or secondary factors (e.g., developmental trajectories, medication exposure), rather than being solely driven by common genetic risk. More recent work by Di Biase et al. [91] extended this framework by demonstrating that cortical thickness deviations in SSD align with distinct cell-type–specific transcriptional profiles, revealing biologically meaningful patient subgroups. These subtypes, characterized by neuronal/endothelial versus glial/OPC signatures, were differentially associated with polygenic risk scores, providing strong evidence that similar clinical phenotypes may reflect fundamentally different cellular and molecular mechanisms. Such findings support the interpretation that extreme deviations identified through normative modeling may index distinct neurobiological “routes” to schizophrenia rather than noise around a single disease process. Finally, longitudinal and stage-sensitive evidence from Zhao et al. [70] suggests that heterogeneity may further evolve across illness stages, with more pronounced cortical thinning and accelerated age-related changes emerging in long-term SSD. This temporal dimension underscores that extreme deviations likely reflect both early neurodevelopmental differences and later illness-related or progressive processes.

Taken together, these findings challenge the notion of SSD as unitary brain disorders and instead support a model in which overlapping clinical phenomenology emerges from diverse and partially independent neurobiological alterations. Within this context, normative modeling offers a powerful framework to capture individual-level deviations that are obscured by traditional case–control approaches. Rather than identifying a single “SSD brain signature,” such approaches may enable the identification of biologically informed subgroups and personalized brain–behavior relationships, with important implications for precision psychiatry and biomarker discovery.

### Strengths and limitations

This study leveraged the PCNtoolkit across multiple independent cohorts, demonstrating its robustness and adaptability to diverse datasets. The leave-site-out CV approach validated the tool’s capacity for generalization, achieving results comparable to prior smaller-scale studies. Importantly, the identification of key age-dependent neuroanatomical features, such as total GMV and superior frontal sulcus, provided meaningful insights into the structural alterations in SSD. Additionally, the utilization of simple ML models, i.e., RF with almost standard hyperparameter settings further highlights a potential replication in future studies. The multivariate analysis further enhanced the understanding of brain-behavior relationships, offering a more integrated perspective on the disorder. Lastly, this is the first study that investigates the NSS and normative deviations of brain structures in different cohorts of SSD patients. The NSS as a neurodevelopmental marker in SSD patients was discussed in previous studies [92, 93]. In this study we could show that the various NSS domains are first, positively connected to brain aging markers and DOI revealed by a multivariate analysis, and second that SSD predictive normative deviations are significantly correlated to NSS total and subscale scores.

Despite these strengths, the classification accuracy of 65%, while above chance, remains insufficient for clinical applications. Misclassification of SSD patients as HC reflects the challenge of capturing the heterogeneity of SSD’s neuroanatomical patterns. Our study analyzed SSD patients from both inpatient and outpatient services, encompassing a broad spectrum of illness stages, durations, and symptom severities. This diversity in demographic and clinical profiles may partly explain why our findings differ from those observed in more homogeneous samples reported in other studies. Second, focusing on one imaging modality can be reductive and the inclusion of functional imaging or diffusion tensor imaging (DTI), could be beneficial for a generalizable classification across cohorts. Rutherford et al. [23] also provided NM for functional connectivity in resting-state fMRI and showed that these features are also able to predict SSD. It was additionally shown that normative deviations derived from DTI measurement can enhance the classification of SSD [24]. It is possible that this multimodal approach could yield the best result in reliably classifying SSD. Finally, although our in-house datasets provided detailed phenotypic information on the psychopathological, cognitive, and sensorimotor domains of SSD patients, this level of granularity is lacking in publicly available datasets. A validation using an independent, deeply phenotyped external cohort would have strengthened the robustness of our findings. Because cognitive testing and NSS were not performed in the HC group, it remains unclear whether the cognitive–motor profile observed in SSD patients is also present in healthy individuals. Future research efforts should focus on expanding and integrating well-characterized SSD cohorts, enabling more comprehensive analyses of the interplay between brain structure, cognition, and sensorimotor function in the disorder.

### Conclusion

This multi-site Identified key predictive features, including GMV changes and ventricle enlargement, that shed light on the SSD’s pathophysiology and link structural deviations to functional outcomes, particularly in cognition and sensorimotor performance. Despite modest classification accuracy and considerable heterogeneity across individuals, these findings highlight the merit of NM in capturing interindividual variability—offering a more nuanced understanding of structural pathology. Our study underscores the value of the PCNtoolkit for elucidating age-dependent neuroanatomical and cognitive-behavioral alterations in SSD. This approach holds promise for advancing precision psychiatry through the identification of reliable biomarkers that may ultimately refine diagnostic accuracy and guide personalized therapeutic interventions. Collectively, these results emphasize the pressing need to further refine neuroimaging methodologies, integrate multimodal data, and employ advanced analytical frameworks to enhance both our mechanistic understanding of SSD and the clinical utility of neuroimaging in psychiatric practice.

## Data Availability

The data that support the findings of this study are available from the corresponding author, upon reasonable request.
